# Entropy Ratio and Entropy Concentration Coefficient, with Application to the COVID-19 Pandemic

**DOI:** 10.3390/e22111315

**Published:** 2020-11-18

**Authors:** Christoph Bandt

**Affiliations:** Institute of Mathematics, University of Greifswald, 17487 Greifswald, Germany; bandt@uni-greifswald.de

**Keywords:** relative entropy, concentration coefficient, Covid-19, fractal, 62B10, 94A17, 62P10, 28A80

## Abstract

In order to study the spread of an epidemic over a region as a function of time, we introduce an entropy ratio *U* describing the uniformity of infections over various states and their districts, and an entropy concentration coefficient C=1−U. The latter is a multiplicative version of the Kullback-Leibler distance, with values between 0 and 1. For product measures and self-similar phenomena, it does not depend on the measurement level. Hence, *C* is an alternative to Gini’s concentration coefficient for measures with variation on different levels. Simple examples concern population density and gross domestic product. Application to time series patterns is indicated with a Markov chain. For the Covid-19 pandemic, entropy ratios indicate a homogeneous distribution of infections and the potential of local action when compared to measures for a whole region.

## 1. Introduction

In any statistical data analysis, a first step is to determine two parameters that characterize center (mean, median) and variation of the data. For the Covid-19 pandemic, the mean in any region is the case incidence, given by weekly averages of case numbers per capita. How can we measure variation of infection intensity? In abstract terms, we have a basic probability measure *Q*, which expresses population size, and another probability measure *P*, which counts infections. We want to know whether *P* is uniformly distributed with respect to Q, or whether it is concentrated on certain hotspots.

Similar questions are frequently studied in economy, where *P* represents market share, wealth, or income, while the basic measure *Q* again expresses population size. The Gini concentration coefficient *G* is the standard parameter of variation [1]. It has a clear, interpretable scale, with values that are between zero and one. Zero describes totally uniform distribution, and one is approached when there are many actors, while all income or production is concentrated in the hands of one monopolist. Gini’s coefficient was used for spatial patterns in medicine [2], and it is applied here to Covid-19 case numbers. However, it does not really fit the data structure and the fractal character of the infection measure P.

Shannon entropy [3] and the relative entropy introduced by Kullback and Leibler [4] have also been used as measures of uniform distribution and concentration. Their values grow with the size of the data, which makes interpretation difficult. In 1971, their use was rejected by Hart [5], since, for the lognormal distribution, a standard model of income distribution, they lead to the ordinary variance. Fractals were not known at this time. However, already in the 1950s, Rényi [6,7] developed entropy concepts that later provided adequate description of fractal measures [8].

Here, we introduce the entropy ratio *U* which is related to Rényi’s entropy dimension of a measure on a metric space. While Rényi’s theoretical concept is defined as a limit, our parameter *U* is simply a descriptive statistics of the measures P,Q. It may vary with the size of the partition on which data of *P* and *Q* are given. Our entropy concentration coefficient C=1−U has the same scale and interpretation as the Gini coefficient. It can be considered as a version of the Kullback–Leibler distance on multiplicative scale.

Entropy concepts indeed seem to be appropriate to describe the spread of an epidemic since there is a strong analogy with Boltzmann’s classical thermodynamical paradigm. A closed system, say molecules in a box with fixed temperature, will approach in the course of time a final state of maximum entropy, which is characterized by uniform distribution of molecules in state space. Under certain conditions, the distance between the actual distribution and the limit distribution is a relative entropy that can be interpreted as a measure of free energy or available work [9].

In the beginning of an epidemic wave, the virus itself will resist this paradigm by creating a fractal structure of hotspots. But if we let the virus do its job, spots will overlap and their number will increase so fast that uniform distribution is approached soon [10]. Of course, reality is not a closed system, and people do their best to avoid this scenario with various containment and lockdown measures. In the language of physics, external forces drive our system away from equilibrium. In this connection, the interpretation of relative entropy as available work seems to be useful for Covid-19. Local and federal authorities in many countries argue on the right type of containment. Measures for a whole country are easier to implement, while local actions can adapt better to a specific situation. The entropy concentration coefficient as a distance to uniform distribution may help to settle such arguments. For small concentration, general measures are justified. When concentration rises, local action becomes more preferable.

We shall now define our concepts, prove their basic properties and compare with Gini’s coefficient. Although Covid-19 was the motivation for our study, it turns out that entropy ratio and entropy concentration coefficient are appropriate parameters for many other phenomena. For this reason, we consider, in Section 3, two small applications to population density and gross domestic product. Section 4 and Section 5 study product spaces and show how entropy ratios are connected with entropy rate of random processes and entropy dimension of measures in Euclidean space. The entropy ratio does not change when the measures P,Q are replaced by their products $P^{k},Q^{k}$ for some k. We introduce Covid-19 data in Section 6 and study the pandemic in the USA and Germany in Section 7 and Section 8, while using data from Johns Hopkins University and Robert Koch Institut, respectively. The paper is completed with conclusions and Appendix A on the Gini coefficient.

## 2. Basic Concepts

*Independence of intensity and concentration.* We take a finite basic space Ω={1,2,…,n}. In our application, the *n* points are the subregions of a region, for example, states of the USA. On this space, two measures $P_{0}$ and $Q_{0}$ are given. In the example, they count the number of infections and inhabitants in each subregion. $P_{0}\left(\Omega \right)$ and $Q_{0}\left(\Omega \right)$ denote the total number of infections and inhabitants in the region. They are important parameters, and $P_{0}\left(\Omega \right)/Q_{0}\left(\Omega \right)$ is the incidence, or intensity, of the disease in the region.

The parameters of uniformity and concentration should be formally independent of the intensity of the observed phenomenon. For this reason, we divide the measures $P_{0},Q_{0}$ by their total mass $P_{0}\left(\Omega \right),Q_{0}\left(\Omega \right),$ respectively. We obtain two probability measures P,Q. In other words, we obtain numbers

(1) $$p_{1},\ldots ,p_{n}\;and\;q_{1},\ldots ,q_{n}\;with\;\sum_{i=1}^{n}p_{i}=\sum_{i=1}^{n}q_{i}=1.$$

When we have twice or ten times the number of cases in the same proportions, we obtain the same $p_{i}$ and $q_{i}.$ Intensity has been eliminated. In the sequel, *Q* will be the measure that is considered as `uniform distribution’. For this reason, we require $q_{i}>0$ for i=1,…,n. Our standard reference method, the Gini coefficient, is described in the Appendix A of the paper.

*Entropy and relative entropy.* The Shannon entropy [3] of the distribution *P* is

(2) $$H=H\left(P\right)=-\sum_{i=1}^{n}p_{i}\;\log p_{i}.$$

Because 0log0=0, all terms of *H* are non-negative. Thus, H≥0, and H=0 only if $p_{i}=1$ for one i. The relative entropy of *P* with respect to *Q* was defined in [4].

(3) $$D=D\left(P,Q\right)=\sum_{i=1}^{n}p_{i}\;\log\frac{p_{i}}{q_{i}}=-\sum_{i=1}^{n}p_{i}\;\log q_{i}-H.$$

Jensen’s inequality for the logarithm implies that *D* is always positive, except for the case P=Q where it is zero, cf. [11] (Lemma 2.3.1). For this reason, *D* is considered to be a kind of distance from *P* to Q, and is called Kullback-Leibler distance, or Kullback-Leibler divergence, or information gain [7]. Note that *D* is finite since we assumed $q_{i}>0.$ If we would interchange *P* and Q, we would get D=∞ as soon as $p_{i}=0$ for some *i* (that is, no infections in a subregion).

On the one hand, *D* is a measure of non-uniformity or concentration of P. It measures how far the proportions of the $p_{i}$ deviate from those of the $q_{i}.$ On the other hand, *D* does not possess a nice bounded scale: when $p_{i}=1$ and $q_{i}=1/K$ for some i,, then D=logK. As a rule, *D* admits larger values when the number *n* of items increases.

*The entropy ratio.* Instead of D, we recommend to take the entropy ratio

(4) $$U=U\left(P,Q\right)=\frac{\sum_{i=1}^{n}p_{i}\;\log p_{i}}{\sum_{i=1}^{n}p_{i}\;\log q_{i}}=\frac{H(P)}{-\sum_{i=1}^{n}p_{i}\;\log q_{i}}=\frac{H}{H+D}$$

as a measure of uniformity, and the entropy concentration coefficient

(5) $$C=1-U=\frac{D}{H+D}$$

as a measure of non-uniformity or concentration. It turns out that *C* has the same properties as Gini’s concentration coefficient.

**Proposition** **1.** *(Basic properties of entropy ratio and entropy concentration coefficient)*

*(i)*  *0≤U≤1 and 0≤C≤1*
*(ii)*  *U=1 and C=0 if and only if P=Q.*
*(iii)*  *U=0 and C=1 if and only if there is an i with $p_{i}=1.$*
*(iv)*  *The values of U and C do not depend on the base of the logarithm.*

**Proof.**  (i). Because logq<0 for 0<q<1 and $q_{i}>0$ for all i, the denominator of *U* is always positive. Thus U≥0. And U≤1 follows directly from D≥0 (see above). (ii). U=1 is equivalent to D=0, which is equivalent to P=Q. (iii). U=0 means H=0, which happens only if some $p_{i}=1.$ (lv). When we first work with natural logarithm and then change to base b, we have to divide both denominator and numerator by logb. □

## 3. Examples

**Figure 1:**
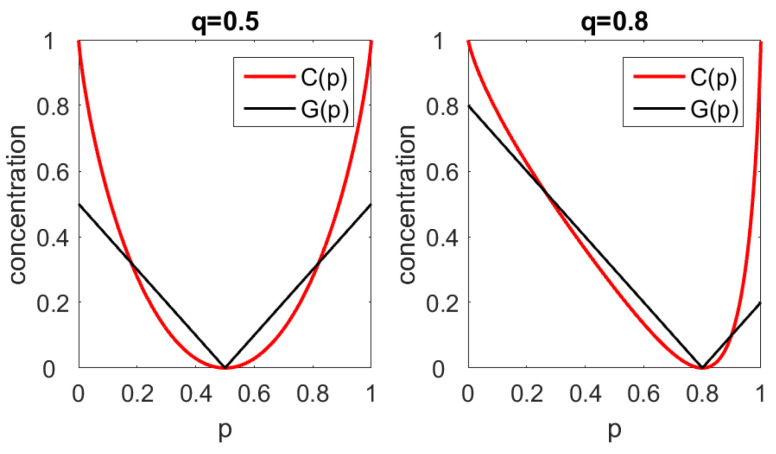
Gini coefficient and entropy concentration coefficient as functions of $p=p_{1}$ for fixed values of q. Left: q=0.5. Right: q=0.8.

*The case of two items.* Let n=2. Setting $p_{1}=p$ and $q_{1}=q,$ a small calculation gives G=|p−q|. For $q=\frac{1}{2}$, we obtain $C=1-p\log_{2}p-\left(1-p\right)\log_{2}\left(1-p\right).$ Figure 1 shows *G* and *C* as functions of *p* while *q* is fixed at $\frac{1}{2}$ and 0.8.

For p=q, we have P=Q, and both coefficients indicate concentration zero. Deviations from this minimum point will be punished stronger by Gini’s coefficient, which is what mathematicians call an $L_{1}$-parameter, cf. [1]. Our coefficient *C* is an $L_{2}$-parameter with a quadratic minimum, which assumes small values in the vicinity of the minimum point.

We see that Gini’s coefficient is not perfect in recognizing monopolists. It approaches the value 1 only if $p_{i}=1$ and also $q_{i}\approx 0.$ Our coefficient *C* will always be 1 for $p_{i}=1,$, as shown in Proposition 1.

*Population density in Germany.* For the 16 federal states and 412 districts of Germany we take the area as $q_{i}$ and the population size as $p_{i}.$ For both states and districts, we show D,G and *C* in Table 1. We want to understand to which extend population is concentrated on small areas.

When compared to the world, Germany has only one large town: Berlin, with 3.6 milliion inhabitants, more than 4000 per ${\mathrm{km}}^{2}.$ It is surrounded by five fairly rural states of East Germany, with a population density of between 70 and 220 people per ${\mathrm{km}}^{2}.$

All three parameters reflect this situation. Deleting Berlin from the statistics must decrease the concentration, and must do so drastically for East Germany. The decrease on the district level is smaller than on state level, since districts of Berlin are well comparable with many urban districts in Germany.

For D, all values of districts are much larger than corresponding values of states. In other words, *D* is biased by sample size n. Moreover, a value of 0.971 does not say anything, since the scale of *D* is unbounded. Accordingly, *D* is rather useless as a concentration measure. The bias of *D* is known, cf. [11] (Lemma 2.3.3), and this paper started as an attempt to standardize D, cf. Proposition 1 (v).

However, *G* is also biased by sample size, with values around 0.5 for districts and around 0.3 for states. According to G, the concentration of districts without Berlin is larger than concentration of states with Berlin. This is simply not true.

The values of *C* in Germany are nearly the same on the state and district level. In the East with Berlin, concentration of districts is clearly smaller than concentration of states, because of the size of Berlin as a state. Without Berlin, the eastern states are very similar, while the districts show a similar structure as all over Germany. On the whole, *C* describes the real situation much better than G. However, in agreement with Figure 1, the values of *C* are generally smaller than those of *G*, since we are fairly near to the minimum point P=Q.

*Gross domestic product.* For all countries with more than one million inhabitants, we took the 2020 population projection from the UN population division [12] and the 2019 gross domestic product (GDP) according to the World Bank database [13] (for a few countries, data of a previous year had to be taken). We study the concentration of the GDP among countries, and among collections of countries defined by the UN as sustainable development goal regions. Table 2 shows the Gini and entropy concentration coefficients determined from these data.

This is a typical application of the Gini coefficient. Nevertheless, *G* is biased by sample size. The values for the world are larger than for the continents, values for regions are smaller than corresponding values for countries. We think that *C* gives a better picture by showing that inequality in Asia is as large as inequality all over the world, while Africa, Europe, and America without US and Canada show a great degree of homogeneity. When the region US+Canada is included, inequality in America becomes very large, due to the immense economic power of the USA.

The values of *C* are again generally smaller than those of G. Hence, *C* may not be welcome as a measure of economic inequality. It takes small deviations from the equality point less seriously than Gini’s G, raising suspicion to downplay social differences.

In the Appendix A, we sketch a proof indicating how *G* depends on sample size. Actually, *G* was not designed for large lists of data of varying length. The standard applications either involve a small number of enterprises that share a market, or an income distribution in classes like 0–1000 $, 1000–4000 $ etc. which is expected to approximate a smooth density function. The geographical distribution of wealth, as measured in regions and subregions, is much more fragmented.

## 4. Product Measures

This note studies measures P,Q which are given by their values on nested partitions. Unfortunately, our data provide only two partitions—states and districts. However, in stochastic processes and time series analysis, we have an unbounded sequence of nested partitions, indexed by words of length 1,2,... over some alphabet. This setting seems to bear a large potential for the application of entropy ratios. We now introduce product measures and show a basic property of the entropy ratio.

If *P* is defined on Ω={1,…,n}, and the probability measure *P* is defined on $\Omega^{\prime }=\left\{1,...,n^{\prime }\right\},$ then the product measure $M=P\times P^{\prime }$ is defined on $\Omega \times \Omega^{\prime }$ by $m_{ij}=p_{i}p_{j}^{\prime }$ for *i* in Ω and *j* in $\Omega^{\prime }.$ The *k*-th power $P^{k}$ is a probability measure on the set $\Omega^{k}$ of all words $w=i_{1}\ldots i_{k}$ of length *k* over the alphabet Ω. The measure $P^{k}$ assigns, to the word *w*, the probability $p_{w}^{k}=p_{i_{1}}p_{i_{2}}\cdot \ldots \cdot p_{i_{k}}.$

Product measures are at the core of entropy theory. When Shannon [3] characterized entropy axiomatically, the following property played a key role.

(6) $$H\left(P\times P^{\prime }\right)=H\left(P\right)+H\left(P^{\prime }\right)\;\mathrm{which implies}\;H\left(P^{k}\right)=k\cdot H\left(P\right).$$

Kullback and Leibler [4] (Theorem 3.1) proved the product formula for relative entropy, and Rényi [7] used it as first axiom for characterizing relative entropy:

(7) $$D\left(P\times P^{\prime },Q\times Q^{\prime }\right)=D\left(P,Q\right)+D\left(P^{\prime },Q^{\prime }\right)\;\mathrm{which implies}\;D\left(P^{k},Q^{k}\right)=k\cdot D\left(P,Q\right).$$

**Proposition** **2.** *(Product invariance of entropy ratio)*

$$U\left(P^{k},Q^{k}\right)=U\left(P,Q\right)\;and\;C\left(P^{k},Q^{k}\right)=C\left(P,Q\right)$$

*for all probability measures P,Q on* Ω *and each positive integer k.*

**Proof.**  The formula directly follows from Proposition 1 (v) and (6),(7), since *k* cancels out. For our finite spaces Ω there is a direct proof by calculation with sums. However, the derivation from (6) and (7) works for arbitrary probability measures. □

It is custom to also consider infinite product measures $P^{\infty },Q^{\infty }$ on the space $X=\Omega^{\infty }$ of all sequences $x=x_{1}x_{2}\ldots x_{k}\ldots$ over the alphabet Ω [3,11]. The basic sets in *X* are the cylinder sets

$$C_{w}=C_{y_{1}...y_{k}}=\left\{x_{1}x_{2}...\in X\;|\;x_{j}=y_{j}forj=1,..,k\right\}.$$

for words $w=y_{1}\ldots y_{k}$ in $\Omega^{k}.$ For each level k, we can consider the partition $P_{k}$ of *X* into the cylinder sets of all words of length k. These partitions form a nested sequence: each $P_{k}$ refines $P_{k-1}.$ Proposition 2 implies

**Proposition** **3.** *(Entropy ratio on infinite products) The measures $P^{\infty },Q^{\infty }$ on the infinite product space X have the same entropy ratio with respect to all partitions $P_{k},k=1,2,...$*

It should be noted that, even in $\Omega^{2},$ the entropy ratio can change if we use other partitions. It is not allowed to split partition sets into small pieces where $p_{i}/q_{i}$ is large, and leaves the cylinder sets big where $p_{i}/q_{i}$ is small. This is known from everyday news: if there are ten reports from districts with more infections than average, and only one report from a district below average, we shall overestimate the danger.

There are various ways to realize infinite product measures in the plane. THe construction of self-similar measures by iterated function systems is a standard method which includes the decimal and the binary number system and can generate fancy fractals [14,15]. The above proposition says that under certain self-similarity assumptions, the entropy ratio of two measures P,Q in the plane will be the same for a sequence of nested partitions.

We do not pretend that a division into states and districts, together with the Covid-19 infection distribution, will fulfil any self-similarity conditions. However, it is natural to expect that the entropy ratios for two nested partitions coincide – in situations where the measure is fractal and the subdivision of the sets of the first partition has nothing to do with the measures P,Q. This is a kind of null hypothesis which says that *P* and *Q* show the same amount of statistical variation on different levels. In reality, there are deviations from the hypothesis, as discussed in Section 6, Section 7 and Section 8.

## 5. Entropy Ratios for Processes and Fractal Measures

To show connections with theory, in this section we consider random processes as in Shannon’s work [3]. Let Ω={0,1} and $q_{0}=q_{1}=\frac{1}{2}.$ Subsequently, $X=\Omega^{\infty }$ is the space of 01-sequences, and $\tilde{Q}=Q^{\infty }$ models sequences of flips of a fair coin. This is the standard uniform distribution: each cylinder set of level *k* has measure $2^{-k}.$ The denominator $D+H=-\sum_{i=1}^{n}{\tilde{p}}_{i}\;\log{\tilde{q}}_{i}$ in the entropy ratio then equals $\log2^{k}=k\cdot \log2$ for the partition $P_{k}$ of *X* into cylinder sets of level k, for any probability measure $\tilde{P}$ on X. In particular, when $\tilde{P}=P^{\infty }$ is a product measure itself, then, for the partition $P_{k}$, we have $U\left(\tilde{P},\tilde{Q}\right)=H\left(P^{k}\right)/\left(k\log2\right)$, which is H(P) with base 2 logarithms, a well-known fact. The limit of $\frac{1}{k}H\left(\tilde{P},P_{k}\right)$ for k→∞ is called entropy rate [3,11]. For finite alphabets, we have

**Proposition** **4.** *(Entropy rate from entropy ratios) Let Q be the equidistribution on a finite alphabet* Ω *and $\tilde{P}$ a probability measure on $X=\Omega^{\infty }.$ Subsequently, the entropy ratios $U(\tilde{P},Q^{\infty })$ on $P_{k}$ are the entropies per letter in words of length k. They converge to the entropy rate of $\tilde{P}.$*

Incidentally, in the first version of this paper, we suggested to take the entropy H(Q) as denominator in order to standardize the relative entropy D. When all partition sets have the same *Q*-measure, then the quantity D/H(Q) agrees with U. Unfortunately, this does not hold in applications: adminstrative districts differ a lot in population size. Therefore, H(Q) was replaced by D+H(P).

Using the correspondence between 01-sequences $x=x_{1}x_{2}\ldots$ and binary numbers $0.x_{1}x_{2}\ldots$, the space $X={\left\{0,1\right\}}^{\infty }$ with its uniform distribution $\tilde{Q}$ can be represented as the unit interval [0,1] with the ordinary length measure. The cylinder sets correspond to intervals $[m2^{-k},\left(m+1\right)2^{-k}].$ The product measure $\tilde{P}$ corresponds to a fractal measure π with dense support in [0,1]. This correspondence is one-to-one, except for a countable set, which has measure zero. We shall see now that the entropy rate of $\tilde{P}$ coincides with the Hausdorff dimension of the measure π.

The entropy dimension of a finite measure μ on $R^{d}$ was defined by Rényi [6] as

$$\dim_{\mathrm{ent}}\mu =\lim_{k\to \infty }\frac{1}{kd\log2}\cdot \sum_{A\in {P^{\prime }}_{k}}\mu \left(A\right)\log\mu \left(A\right)\;,$$

where ${P^{\prime }}_{k}$ denotes the partition of $R^{d}$ into *d*-dimensional dyadic cubes of side length $2^{-k}.$ Here, we have d=1. Obviously, Rényi had in mind the connection between dyadic cubes and 01-words. In 1982, Young proved that the entropy dimension and Hausdorff dimension of a measure coincide if the measure is homogeneous in the sense that pointwise dimension is the same almost everywhere [8] (Section 4). Without going into further detail (see [15] for Hausdorff and pointwise dimension), we just note that on the unit cube, where the volume measure λ is a probability, the term behind the limit is the entropy ratio U(μ,λ). Thus

**Proposition** **5.** *(Entropy ratios approximate fractal dimension)*

*Let μ be a probability measure on ${\left[0,1\right]}^{d}$ which fulfils the homogeneity condition of [8]. Let λ denote the volume measure and ${P^{\prime }}_{k}$ the partition of the unit cube into dyadic cubes of side length $2^{-k}.$ Subsequently, the entropy ratios $U_{k}\left(\mu ,\lambda \right)$ on these partitions converge to the Hausdorff dimension of μ.*

It should be added that a quotient of logarithms, like *U*, is the typical form of a fractal dimension [15]. Great effort was invested in proving the existence of entropy rates and dimension limits [11,15]. Our point here is that, in real-world situations, limits are hard to determine, but the first terms of the sequence have a descriptive power and can be calculated.

Young’s homogeneity condition is fulfilled for the product measures and for the Markov chains that we consider now. We assume that $\tilde{P}$ is generated by a Markov chain, which does not permit the word 11. The transition probabilities are $p_{11}=0,p_{10}=1$ and $p_{01}=t,p_{00}=1-t.$ The stationary distribution is given as $p_{0}=\frac{1}{1+t}$ and $p_{1}=\frac{t}{1+t},$ On the interval, the corresponding measure π lives on those binary numbers that have no two consecutive digits 1. They form a Cantor set $A=A_{0}\cup A_{1}\subset \left[0,\frac{2}{3}\right].$ Here, $A_{0},A_{1}$ denote the subsets of *A* that consist of binary numbers with first digit 0 and 1, respectively. They fulfil the equations

$$A_{0}=f_{0}\left(A_{0}\right)\cup f_{0}\left(A_{1}\right)\;\mathrm{and}\;A_{1}=f_{1}\left(A_{0}\right)\;,\;\mathrm{thus}\;A_{0}=f_{0}\left(A_{0}\right)\cup f_{0}f_{1}\left(A_{0}\right)$$

where $f_{0}\left(x\right)=x/2$ and $f_{1}\left(x\right)=\left(x+1\right)/2$ assign to $0.x_{1}x_{2}\ldots$ the values $0.0x_{1}x_{2}\ldots$ and $0.0x_{1}x_{2}\ldots ,$ respectively.

The similarity maps $f_{0}$ and $f_{0}f_{1}$ have factors $\frac{1}{2}$ and $\frac{1}{4},$ respectively. Accordingly, the Hausdorff dimension α of $A_{0}$ can be determined from the equation ${\left(\frac{1}{2}\right)}^{\alpha }+{\left(\frac{1}{4}\right)}^{\alpha }=1$ [14,15]. We get $\alpha =\log_{2}\tau \approx 0.694$ where τ≈1.618 is the golden mean. For every *t* between 0 and 1, π is a measure on *A*, which has dimension smaller than or equal to α.

The measure π has maximal dimension when t=1/τ, or $t^{2}=1-t.$ In this case π is the normalized Hausdorff measure on A. This is the uniform distribution on A, so it seems clear that it has the smallest distance to the uniform distribution on [0,1], which is, minimal concentration 0.306. In this case, all of the cylinder sets have measure $t^{k}$ for some k, and entropy ratios can be expressed with Fibonacci numbers $F_{k}$ as $U_{k}=\left(F_{k+1}+F_{k}\frac{k+1}{k}t\right)t^{k}\log_{2}\tau .$ For any Markov chain, we can easily determine entropy ratios by matrix calculation, however. Table 3 shows how the concentration values $C_{k}=1-U_{k}$ approach their limit. The first step away from the uniform distribution is decisive, and the convergence slows down after the second step.

It seems interesting to determine similar distances between empirical distributions and model distributions, taking, for instance, time series of order patterns derived from high-frequency biomedical measurements, as in [16].

## 6. The Global Covid-19 Pandemic

We study Covid data for two reasons. On the one hand, even though they are given only on two levels, they give a good testbed, since they are determined every day. We can determine time series of concentration coefficients, and their graphs give much more information than the tables in Section 3. On the other hand, we provide a little tool for the investigation of previous waves of infection which perhaps can help to prevent the next one. We refrain from evaluating countries or political measures. That would require much more care, including study of social and economic aspects.

The numbers of positively tested cases and deaths are taken as seven-day sums per 100,000 inhabitants of the region. The data come from the database of Johns Hopkins University [17]. Weekly averages smooth out random variations of the daily numbers as well as systematic variations caused by weekends where administrations have no office hours. Sums are taken instead of averages, since seven days is the typical period for which an infected person remains infectious [18]. Thus, the sum over one week is a measure of the infection potential or Covid activity in a region. Such sums have become the basis of government decisions in Germany [19], and a main criterion for defining hotspots of the pandemic in the USA [20]. The seven-day sums are updated every day, which results in a daily time series for each country and province. See [17,21] for more details.

**Figure 2:**
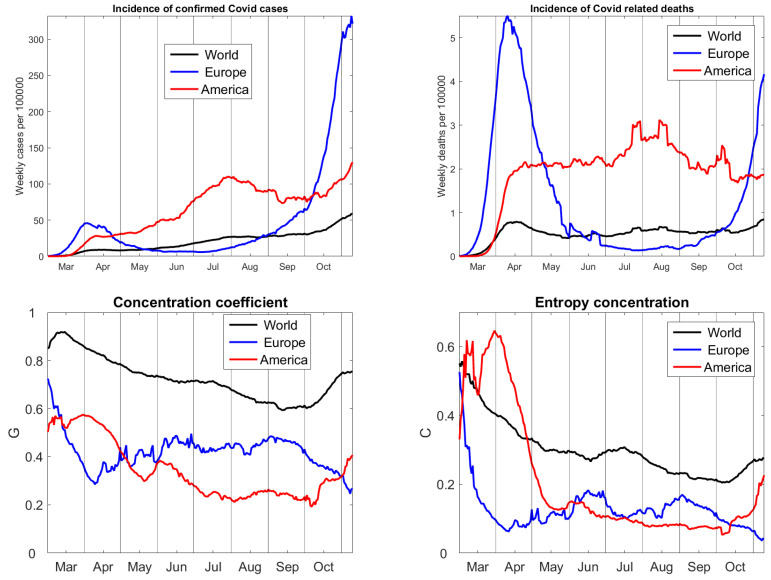
Time series of the pandemic. The upper row describes the intensity of the pandemic, with cases on the left and deaths on the right. The lower row specifies the non-uniformity of the distribution of cases, with Gini coefficients on the left and entropy concentration coefficients on the right. Data from Johns Hopkins University [17], updated 10 November 2020.

Figure 2 shows a global view, summarizing the values of 55 large countries, including 23 from Europe, three from North, and six from South America. The number of cases on the whole world is continuously increasing, with slow growth in August and September. The worldwide number of deaths remained almost constant at about six Covid-related deaths per one million people and week. In Europe, there was a wave of infections in March and April, and, now, there is a much larger second wave of cases. The first wave came with large numbers of deaths which lasted only for few weeks. The death rate of the second wave is now twice as large as in America. The number of cases in America increased from March to July, declined in August and is rising again. The number of deaths in America remained on a level four times as large as the world’s average for a long time, higher in July and August, and lower in October. The two rectangular peaks come from irregularities in the data, when a statistical office reported one thousand more deaths on a single day which they did not count before. We did not smooth the data. Because we take seven-day sums, irregularities of a day remain in the time series for 13 days. As a matter of fact, errors, like negative daily numbers of deaths, appear quite frequently in Europe (for instance, France, Spain, Germany) and in the USA, but not in Brazil or Iran.

Time series of concentration coefficients were calculated for cases only. For deaths, they look similar, but more distorted due to the irregularities in the data. Gini’s *G* and our *C* show a similar time course. The pandemic becomes increasingly spread over the world, but the concentration increased since October because of the second wave in Europe. In Europe, raise of concentration from April to June indicated desrease of cases in various countries, while the second wave now hits all of Europe, causing uniform distribution of infections. In America, the situation has been constant for several months, but since October the concentration rises, probably due to an improvement in Brazil and Peru and increase of cases in Argentina and the US.

At second glance, we recognize the sample size bias of G. The worldwide concentration is large because of Asian countries, like China, Vietnam, and Thailand, which have almost no cases. However, it is not as large as indicated by G. The difference between Gini concentration levels of America and Europe is too large and seems to be caused by the fact that we had 23 countries from Europe and only nine from America. The American values have less variation, when compared with the world and Europe. Therefore, the initial concentration of the pandemic in the north-east of the US in March/April is underestimated. For entropy concentration, the variation is larger and the initial concentration in New York is more correct. For the world, the initial maximum concentration (without counting Wuhan) was rather in the beginning of March, as shown by C, than in the middle of March, as indicated byG. Altogether, entropy concentration gives a more correct description than Gini’s coefficient. However, the values of *C* are considerably smaller than those of G.

## 7. States and Counties in the USA

**Figure 3:**
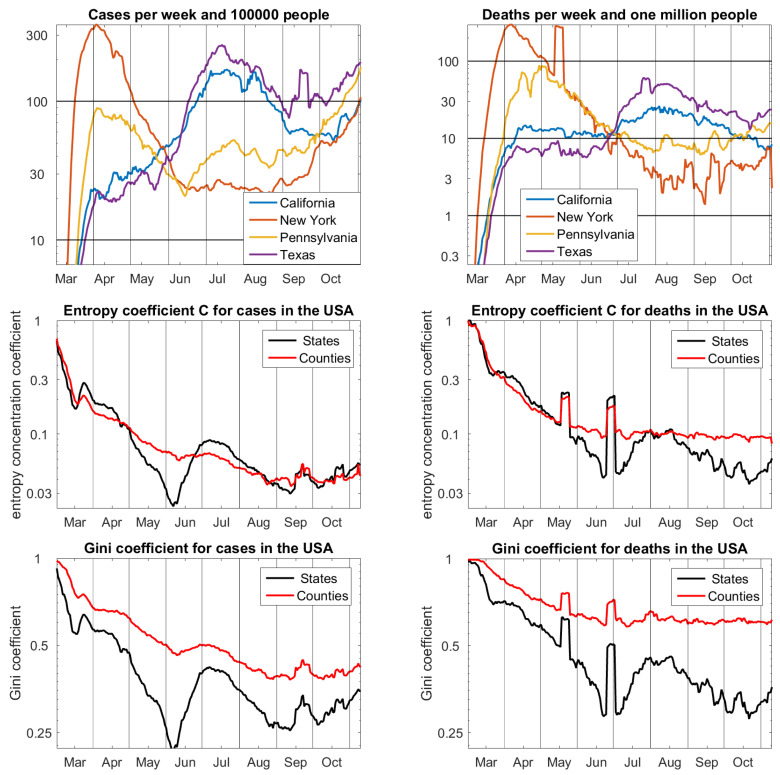
**Upper row**: cases and deaths for four representative states of the USA. **Middle row**: entropy concentration coefficients for the partitions into 51 states and 3143 counties on logarithmic scale. **Lower row**: Gini coefficients are very similar, but larger and not adapted to sample size. Data from Johns Hopkins University [17], 10 November 2020.

The most comprehensive public dataset of the pandemic, maintained by the Johns Hopkins University, contains case and death numbers of 3143 counties of the 51 federal states of the USA. For Figure 3, we have chosen four states that represent the time development. New York suffered a lot in the beginning of the pandemic and has now shown the smallest numbers of cases for some months. Pennsylvania had a similar development, less dramatic in the beginning, and with more recent infections. In California and Texas, the peak of the pandemic came in summer. Curiously, all four, and a number iof other states, had approximately the same number of cases in the beginning of June, and the same number of deaths in the end of June. This is reflected by the concentration coefficients.

In March, the pandemic first concentrated near Seattle and then in New York and the northeast of the US, which results in concentration values that are near one. For this reason, *C* and *G* are shown by logarithmic scale on [0.022,1] and [0.22,1], respectively. With this modification, it is surprising how similar *C* and *G* are as functions of time. Once again, it becomes apparent how much *G* is pushed by sample size. The picture of *C* with values of the same magnitude for states and counties seems to be correct. The concentration values for deaths in counties look almost constant since June. They are greater than corresponding values of states and greater than values for cases. Different conditions of cities and countryside could be a reason. Among others, large intensive care units are usually in cities. The two peaks in the concentration functions for deaths come from data irregularities in New York and New Jersey, respectively. Because these were spread over various counties in one state, they disturb the function of states more than the function of counties.

## 8. States and Districts in Germany

We use the case-related public file RKI COVID19 [22] of Robert Koch Institut, the health authority in charge of epidemic. These data allow for determining accurate case fatality rates, the distribution of daily cases in 16 federal states and 412 districts, and their joint distribution with six age classes.

**Figure 4:**
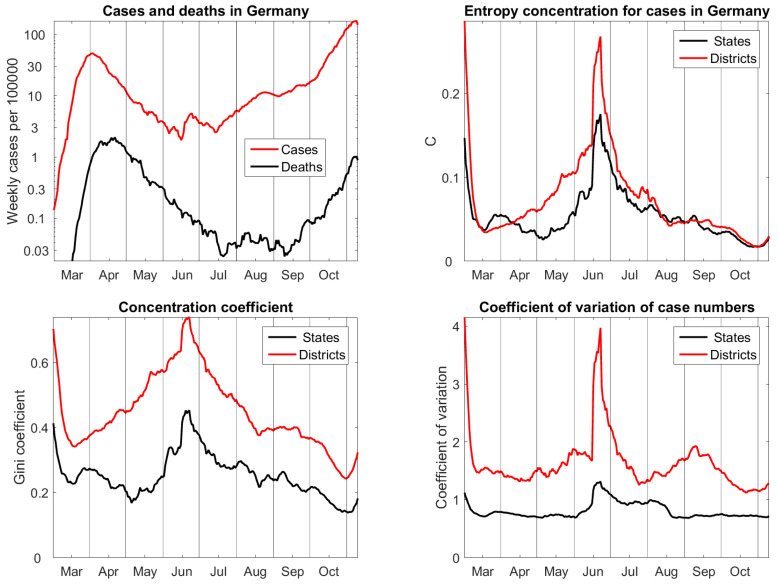
**Upper left**: cases and deaths in Germany. **Upper right**: entropy concentration coefficients for the 16 federal states and 412 districts. **Lower row**: Gini coefficient and coefficient of variation are biased by sample size. Data from Robert Koch Institut [22], updated 10 Nov 2020.

Figure 4 shows the cases and deaths on a logarithmic scale. Germany is one of the European countries with mild impact of the first Covid-19 wave. The case fatality rate was about 5% in April and then decreased tremendously, below 0.5%. In June, the cases reached a minimum. Just at this time, there was a big outbreak in the main meat enterprise, which caused the big peak in concentration functions. Since July, cases have been continuously rising, and deaths followed since September.

The entropy concentration coefficients for states and districts do almost coincide since July, and they show a continuous approach to uniform distribution, with exception of the very last week. However, in spring, the concentration of districts was higher than concentration of states. This is true, because many districts with small population density recovered very fast from the first wave, so that for several weeks there were between 100 and 200 districts each day without cases. On state level, the differences were smaller. The outbreak in the meat factory appeared in the federal state with the largest population, but in a district of average size that explains why the peak is so much bigger for districts than for states.

Gini coefficients show similar features as the entropy concentration coefficients C, but their bias on sample size is obvious. In the search for concentration measures we also calculated standard deviations of case numbers or probabilities $p_{i}$ with respect to the weights $q_{i}$ They gave no reasonable result, no matter whether we took ordinary or logarithmic scale. The only variant worth mentioning is given by coefficients of variation of the case numbers (for the $p_{i}$ the parameter is the same). That is, the standard deviation divided by mean. Figure 4 shows that they admit no natural scale and have a bias. Actually they do not show much change in time, except for the meat factory outbreak. The parameter *C* clearly performs the best, and our null hypothesis of equal concentration for the two partitions is confirmed by the remarkable coincidence of the two time series.

**Figure 5:**
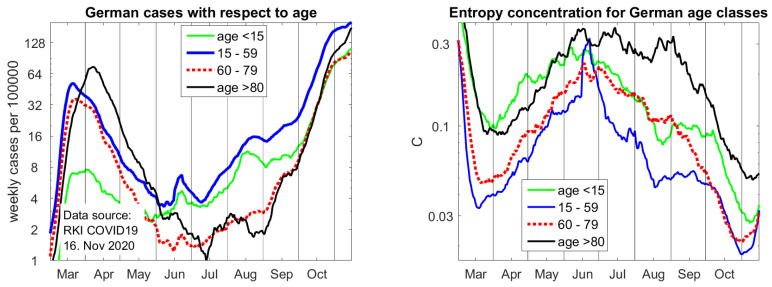
**Left**: case incidence with respect to age. **Right**: entropy concentration coefficients of cases for age groups. These figures were updated in proof on 16 November since the most recent concentration coefficients already indicate improvement in part of the country while case incidences are still rising.

Because Covid deaths are the most frequent among old people, it is very important to study the age dependence of cases, as shown on the left of Figure 5. There are four age classes: children below 15, the working population in the age of 15 up to 59, the elderly with 60 to 79 years, who have case fatality rate of about 2%, and the very vulnerable group of age 80 and above, who now have a case fatality of about 12% (in spring, fatality rates were much higher since even among old people, only cases with serious symptoms were tested). The last group, represented by nearly six-million inhabitants of Germany, needs the most protection.

Until August, the seven-day incidence of people above 80 was very low, only 2 per 100,000. In September, the number raised to eight and, on November 10, it is 138. Simple calculation shows that this is more or less the number of deaths in this group, which we can expect all over Germany two weeks later. Approximately 60% of all Covid deaths come from this age class. So it is possible to predict deaths in Germany two weeks in advance. It should be noted that the rise of infections of old people is not due to their careless behavior or lack of protection. More likely, the number of infections have risen in all age classes, but younger people do not easily get tested anymore.

For our study, it is interesting to consider the concentration functions *C* in different age classes. We use the districts that give more information. It can be seen that cases in the group of working age 15–59 are most uniformly spread, certainly due to their mobility. The outbreak in the meat factory is almost restricted to this group. The infections in the group of children and the group of elderly above 80 were more concentrated in certain districts. However, with the progress of the second wave, they have also become uniformly spread. While local action was perhaps still good in September, it is quite reasonable that a partial lockdown for all Germany took effect on November 2. There is an upwards trend in the most recent concentration coefficients, which indicates that, like in March, districts with a small population density are already improving. However, incidences are still growing.

## 9. Conclusions

We intended to describe the spread of the Covid-19 pandemic, given by daily numbers of confirmed cases and deaths in various regions. The question is how uniform the distribution of infections is with respect to the distribution of population. The Kullback–Leibler distance can be applied, but it is biased by sample size and has an unbounded scale. The well-established Gini coefficient can be used and it has a unit scale, but it is strongly influenced by sample size. It was not designed for data sampled in many regions and districts. When countries are compared by Gini coefficients, with respect to the inequality of income say, then a smooth income distribution is calculated for every country. The Covid data structure of cases and deaths reflects the spatial distribution of infections, which is more fragmented. Many variables can be sampled in this way: air and water pollution, precipitation, wealth, crime, etc.

An entropy concentration coefficient *C* was introduced. It has the same scale and basic properties as the Gini coefficient and it seems to be much less dependent on sample size. The new coefficient is a multiplicative version of the Kullback–Leibler divergence and can be interpreted as distance of a probability measure *P* from a uniform distribution. When the uniform distribution is volume in Euclidean space, and *P* is homogeneous, then *U* approximates the Hausdorff dimension of P. When the uniform distribution is the product measure of an equidistribution on a finite alphabet, then U=1−C is connected with the entropy rate. In the setting of random processes and empirical distributions of long time series, we anticipate applications of C.

We analysed data of the global pandemic, of counties in the US and of districts in Germany. The concentration coefficient *C* as a function of time becomes small when infections become widespread. Its growth can indicate improvement, sometimes earlier than the decline of cases. Large values of *C* also appear in the initial stage of an epidemic, and for big local outbreaks. In Germany, age groups—in particular, the vulnerable group of elderly above 80—develop differently with respect to concentration. A referee suggested that cross-correlation between countries and age classes could be further studied by forecasting and backcasting analysis, while using detrended cross-correlation coefficients, as in [23]. All in all, the entropy concentration coefficient is an interesting new parameter.

## Figures and Tables

**Table 1:** Concentration of population in German states and districts.

Parameter	Germany	without Berlin	East with Berlin	East without Berlin
*D* for states	0.260	0.184	0.618	0.089
*D* for districts	0.650	0.580	0.971	0.469
*G* for states	0.336	0.309	0.390	0.227
*G* for districts	0.528	0.509	0.539	0.414
*C* for states	0.098	0.073	0.262	0.054
*C* for districts	0.101	0.092	0.183	0.101

**Table 2:** Concentration of gross domestic product in countries and regions.

Parameter	World	Asia	Africa	Europe	America	without US, Ca
number of regions/countries	19/159	5/47	5/49	4/35	4/25	3/23
*G* for regions	0.575	0.379	0.291	0.282	0.441	0.012
*G* for countries	0.621	0.496	0.412	0.340	0.471	0.190
*C* for regions	0.218	0.210	0.092	0.102	0.382	0.0004
*C* for countries	0.182	0.177	0.088	0.067	0.269	0.034

**Table 3:** Concentration of Markov Fibonacci measure on words of length *k*.

*k*	1	2	3	4	5	6	*∞*
$C_{k}$	0.0406	0.2382	0.2588	0.2671	0.2739	0.2783	0.30576
$C_{k}-C_{k-1}$		0.1977	0.0206	0.0083	0.0068	0.0044	

## Appendix A. The Gini Coefficient

*Definition of G.* Given the $p_{i},q_{i}$ in (1), we renumber the *n* items in such a way that $p_{1}/q_{1}<p_{2}/q_{2}<...<p_{n}/q_{n}.$ For an epidemic, this means ordering subregions by increasing incidence. To save notation, we assume the renumbering has already been done. Now, take the cumulative probabilities $x_{k}=\sum_{i=1}^{k}q_{i}$ and $y_{k}=\sum_{i=1}^{k}p_{i}$ for k=1,...,n. Let $\left(x_{0},y_{0}\right)=\left(0,0\right)$ and note that $\left(x_{n},y_{n}\right)=\left(1,1\right).$ The polygonal line which connects the successive points $(x_{k},y_{k}),k=0,1,...,n$ in the xy-plane is called Lorenz curve. Due to the assumption of increasing quotients, the Lorenz curve runs below the line y=x. The Gini coefficient *G* is twice the area between Lorenz curve and line y=x. For details, see any statistics textbook for economists or [1].

Now, we show that the Gini coefficient increases when the partition determining the $p_{i},q_{i}$ is refined. It is enough to prove this for a basic step of refinement, where one partition set is subdivided into two. It would be interesting to modify the geometric proof so that under stochastic model assumptions the increase can be quantified. The area described below can easily be calculated.

**Proposition** **A1.**
*(Gini coefficient increases under refinement)*

*Let G be the Gini coefficient for probabilities $p_{i},q_{i}$ with $p_{i}/q_{i}<p_{i+1}/q_{i+1}$ for i=1,...,n. Now suppose that one pair of probabilties is splitted: $p_{k}=p_{k}^{\prime }+p_{k}\prime \prime$ and $q_{k}=q_{k}^{\prime }+q_{k}\prime \prime .$ Then the new Gini coefficient $G^{\prime }$ is larger than G, except for the case $p_{k}^{\prime }/q_{k}^{\prime }=p_{k}\prime \prime /q_{k}\prime \prime$ where $G^{\prime }=G.$*

**Proof.** We can assume $p_{k}^{\prime }/q_{k}^{\prime }\leq p_{k}\prime \prime /q_{k}\prime \prime .$ For the Lorenz curve connecting the points $\left(x_{i},y_{i}\right)$ defined above, we add one more vertex (x,y) with $x=\sum_{i<k}q_{i}+q_{k}^{\prime }$ and $y=\sum_{i<k}p_{i}+p_{k}^{\prime }.$ When $p_{k}^{\prime }/q_{k}^{\prime }=p_{k}\prime \prime /q_{k}\prime \prime$ then (x,y) is on the Lorenz curve and $G^{\prime }=G.$ Otherwise, (x,y) is below the Lorenz curve, and connecting the points with increasing x, the area between the new Lorenz curve and the line y=x is enlarged by the triangle with vertices $A=\left(x_{k-1},yk-1\right),B=\left(x,y\right),$ and $C=(x_{k},y_{k}).$ However, in that case, we have to reorder the points, which will lead to even more enlargement. To save notation, we only consider one step of reordering. We assume that $p_{k-1}/q_{k-1}>p_{k}^{\prime }/q_{k}^{\prime }$ so that $k^{\prime }$ and k−1 must interchange order. In that case the point *A* of the Lorenz curve is replaced by $A^{\prime }=\left(x_{k-2},y_{k-2}\right)+\vec{AB},$ and the parallelogram with vertices $A,B,A^{\prime },\left(x_{k-2},y_{k-2}\right)$ will be added to the area between Lorenz curve and line y=x. When $k^{\prime }$ has to be arranged still further down, then more of these parallelograms will be added. The same holds for ordering k″ above k. Those parallelograms are constructed with vector $\vec{CB}$ instead of $\vec{AB}.$ The more $p_{k}^{\prime }/q_{k}^{\prime }$ and $p_{k}\prime \prime /q_{k}\prime \prime$ differ, the more parallelograms have to be added in order to obtain $G^{\prime }$ from G. □
